# Supplementary material for: JAK2 Unmutated Polycythaemia—Real-World Data of 10 Years from a Tertiary Reference Hospital
Source: J Clin Med. 2022 Jun 13;11(12):3393. doi: 10.3390/jcm11123393 (PMC9225037; doi:10.3390/jcm11123393)
Supplement: Supplementary file 1 [file jcm-11-03393-s001.zip › jcm-1750073-supplementary.pdf]

Table S1. Therapy of JAK2 unmutated polycythaemia patients, N=294.

Type of Polycythaemia	N, All	Age, Median Years (Range)	Therapy Received, N=61						Therapy Ongoing, N=22				
			N	P+ASS, N	P, N	ASS, N	P+ASS+IFN, N	P+ASS+HU	N	P+ASS, N	P, N	ASS, N	P +IFN, N
**All**	**294**	**46 (15, 89)**	**61**	**11**	**42**	**6**	**1**	**1**	**22**	**4**	**9**	**8**	**1**
**1. Relative**	**14**	**55 (22, 70)**	**5**	**2**	**3**	**0**	**0**	**0**	**1**	**0**	**0**	**1**	**0**
1.1. Normal RBC mass	9	59 (25, 68)	5	2	3	0	0	0	1	0	0	1	0
1.2. Dehydration (clinical diagnosis)	1	22 (22, 22)	0	0	0	0	0	0	0	0	0	0	0
1.3. Gaisböck syndrome	2	49 (35, 63)	0	0	0	0	0	0	0	0	0	0	0
1.4. Capillary leak syndrome	2	54 (37, 70)	0	0	0	0	0	0	0	0	0	0	0
**2. Reactive oxygen driven**	**139**	**47 (17, 89)**	**23**	**4**	**16**	**3**	**0**	**0**	**7**	**0**	**4**	**3**	**0**
2.1. Sleep apnoea	55	49 (20, 89)	13	3	8	2	0	0	4	0	2	2	0
2.2. HbCO >5%	14	52 (18, 69)	2	0	2	0	0	0	1	0	1	0	0
2.3. Altitude	1	43 (43, 43)	0	0	0	0	0	0	0	0	0	0	0
2.4. Smoking	51	45 (18, 71)	6	1	4	1	0	0	2	0	1	1	0
2.5. Respiratory disease	13	47 (17, 72)	2	0	2	0	0	0	0	0	0	0	0
2.6. Cardiac disease	5	40 (21, 50)	0	0	0	0	0	0	0	0	0	0	0
**3. Reactive Epo/hormonal driven**	**40**	**55 (23, 82)**	**6**	**1**	**5**	**0**	**0**	**0**	**2**	**1**	**1**	**0**	**0**
3.1. Increased Epo	4	68 (31, 76)	1	0	1	0	0	0	0	0	0	0	0
3.2. Non-cancer kidney disease	12	52 (23, 76)	2	1	1	0	0	0	2	1	1	0	0
3.3. Post-renal transplantation	7	59 (32, 69)	1	0	1	0	0	0	0	0	0	0	0
3.4. Androgen therapy	9	50 (25, 69)	2	0	2	0	0	0	0	0	0	0	0
3.5. Cancer (renal, adrenal, seminoma, lung, brain)	8	68 (32, 82)	0	0	0	0	0	0	0	0	0	0	0
**4. Congenital**	**12**	**33 (16, 71)**	**4**	**0**	**3**	**0**	**0**	**1**	**3**	**0**	**3**	**0**	**0**
4.1. Mutations/hemoglobinopathy	4	29 (22, 34)	3	0	3	0	0	0	3	0	3	0	0
4.2. Down's syndrome	2	37 (16, 57)	0	0	0	0	0	0	0	0	0	0	0
4.3. High affinity Hb (increased P50)	6	45 (28, 71)	1	0	0	0	0	1	0	0	0	0	0
**5. Undetermined (idiopathic) (group 5)**	**89**	**43 (15, 77)**	**23**	**4**	**15**	**3**	**1**	**0**	**9**	**3**	**1**	**4**	**1**

Abbreviations: P – phlebotomy (=venesection), ASS – Aspirin, HU – Hydroxyurea, IFN – Interferon.

Table S2. Thrombotic events during follow-up in JAK2 unmutated polycythaemia patients, N=294.

**Type of Polycythaemia**	**N, all**	**Age, Median Years (Range)**	**All Thrombotic Events, N=65**	**Venous Thrombotic Events, N=33**						**Arterial Thrombotic Events, N=32**			
				**DVT**	**Splanchnic**	**PE**	**Ophthalmic**	**Cerebral**	**Peripheral**	**Cardiac**	**Stroke**	**MI**	**Peripheral**
**All**	**294**	**46 (15, 89)**	**65**	**10**	**2**	**16**	**1**	**1**	**2**	**1**	**16**	**11**	**5**
**1. Relative**	**14**	**55 (22, 70)**	**6**	**0**	**0**	**0**	**0**	**0**	**1**	**1**	**1**	**0**	**3**
1.1. Normal RBC mass	9	59 (25, 68)	0	0	0	0	0	0	0	0	0	0	0
1.2. Dehydration (clinical diagnosis)	1	22	3	0	0	0	0	0	0	0	0	0	3
1.3. Gaisböck syndrome	2	49 (35, 63)	3	0	0	0	0	0	1	1	1	0	0
1.4. Capillary leak syndrome	2	54 (37, 70)	0	0	0	0	0	0	0	0	0	0	0
**2. Reactive oxygen driven**	**139**	**47 (17, 89)**	**32**	**6**	**0**	**11**	**0**	**0**	**1**	**0**	**6**	**6**	**2**
2.1. Sleep apnoea	55	49 (20, 89)	15	3	0	3	0	0	0	0	5	2	2
2.2. HbCO >5%	14	52 (18, 69)	0	0	0	0	0	0	0	0	0	0	0
2.3. Altitude	1	43	0	0	0	0	0	0	0	0	0	0	0
2.4. Smoking	51	45 (18, 71)	10	3	0	3	0	0	0	0	0	4	0
2.5. Respiratory disease	13	47 (17, 72)	5	0	0	5	0	0	0	0	0	0	0
2.6. Cardiac disease	5	40 (21, 50)	2	0	0	0	0	0	1	0	1	0	0
**3. Reactive Epo/hormonal driven**	**40**	**55 (23, 82)**	**7**	**3**	**1**	**1**	**0**	**0**	**0**	**0**	**1**	**1**	**0**
3.1. Increased Epo	4	68 (31, 76)	1	1	0	0	0	0	0	0	0	0	0
3.2. Non-cancer kidney disease	12	52 (23, 76)	1	0	0	0	0	0	0	0	0	1	0
3.3. Post-renal transplantation	7	59 (32, 69)	1	1	0	0	0	0	0	0	0	0	0
3.4. Androgen therapy	9	50 (25, 69)	2	1	0	0	0	0	0	0	1	0	0
3.5. Cancer (renal, adrenal, seminoma, lung, brain)	8	68 (32, 82)	2	0	1	1	0	0	0	0	0	0	0
**4. Congenital**	**12**	**33 (16, 71)**	**3**	**0**	**0**	**1**	**0**	**0**	**0**	**0**	**1**	**1**	**0**
4.1. Mutations/hemoglobinopathy	4	29 (22, 34)	0	0	0	0	0	0	0	0	0	0	0
4.2. Down's syndrome	2	37 (16, 57)	1	0	0	1	0	0	0	0	0	0	0
4.3. High affinity Hb (increased P50 high)	6	45 (28, 71)	2	0	0	0	0	0	0	0	1	1	0
**5. Undetermined (idiopathic)**	**89**	**43 (15, 77)**	**17**	**1**	**1**	**3**	**1**	**1**	**0**	**0**	**7**	**3**	**0**

Abbreviations: DVT – deep venous thromboembolism, PE – pulmonary embolism, MI – myocardial infarction.
